# Detection of Cancer-Associated Gene Mutations in Urinary Cell-Free DNA among Prostate Cancer Patients in South Africa

**DOI:** 10.3390/genes14101884

**Published:** 2023-09-27

**Authors:** Dada Oluwaseyi Temilola, Martha Wium, Juliano Paccez, Azola Samkele Salukazana, Solomon O. Rotimi, Hasan H. Otu, Giuseppina M. Carbone, Lisa Kaestner, Stefano Cacciatore, Luiz Fernando Zerbini

**Affiliations:** 1 International Centre for Genetic Engineering and Biotechnology (ICGEB), Cape Town 7925, South Africa; dada.temilola@icgeb.org (D.O.T.); mariet.wium@icgeb.org (M.W.); juliano.paccez@icgeb.org (J.P.); stefano.cacciatore@icgeb.org (S.C.); 2 Integrative Biomedical Sciences Division, Faculty of Health Sciences, University of Cape Town, Cape Town 7925, South Africa; 3 Division of Urology, University of Cape Town, Groote Schuur Hospital, Cape Town 7925, South Africa; 203samec@gmail.com (A.S.S.); lisalisa@live.co.za (L.K.); 4 Biochemistry Department, Covenant University, Ota 112104, Nigeria; ola.rotimi@covenantuniversity.edu.ng; 5 Department of Electrical and Computer Engineering, University of Nebraska-Lincoln, Lincoln, NE 68588, USA; hotu2@unl.edu; 6 Institute of Oncology Research (IOR), Università della Svizzera italiana, 6900 Bellinzona, Switzerland; giuseppina.carbone@ior.usi.ch

**Keywords:** cfDNA, prostate cancer, South Africa, WGS, SNVs

## Abstract

Prostate cancer (PCa) is the most common cause of cancer death among African men. The presence of tumor-specific variations in cell-free DNA (cfDNA), such as mutations, microsatellite instability, and DNA methylation, has been explored as a source of biomarkers for cancer diagnosis. In this study, we investigated the diagnostic role of cfDNA among South African PCa patients. We performed whole exome sequencing (WES) of urinary cfDNA. We identified a novel panel of 31 significantly deregulated somatic mutated genes between PCa and benign prostatic hyperplasia (BPH). Additionally, we performed whole-genome sequencing (WGS) on matching PCa and normal prostate tissue in an independent PCa cohort from South Africa. Our results suggest that the mutations are of germline origin as they were also found in the normal prostate tissue. In conclusion, our study contributes to the knowledge of cfDNA as a biomarker for diagnosing PCa in the South African population.

## 1. Introduction

Prostate cancer (PCa) is the most common cancer among males, resulting in over 1,193,715 new cases and 375,000 fatalities each year [1]. In African men, PCa presents more aggressively [2]. Yet, it remains uncertain whether this increased aggression is an inherent biological characteristic found in African patients, as noted in African-descent migrant populations, or is a result of late-stage diagnosis, which could affect disease treatment. Several studies have been carried out to comprehend the diverse genomic [3], proteomic [4,5], miRNA [6], and metabolomic [7] profiles of African PCa patients in an attempt to understand the underlying disease pathology. Such a genomics approach has been used to predict disease aggression in men of African descent with localized PCa [8].

The Gleason score is a measurement of PCa aggressiveness and is based on the histological features of the prostate tissue. Higher scores are associated with increasing cell abnormalities and dysregulation of cellular processes implicated in cancer progression. The shift from well-differentiated to poorly differentiated cells signifies key pathophysiological alterations that fuel cancer’s growth, including genetic mutations, angiogenesis, hormonal fluctuations, invasion, and metastasis. Additionally, prostate-specific antigen (PSA), a blood base biomarker, is used for PCa screening [9]. Unfortunately, PSA has a low specificity [10]; thus, the diagnosis must be confirmed through needle biopsies that have side effects such as discomfort and pain. PSA screening often leads to overdiagnosis of clinically insignificant PCa and consequent overtreatment [11]. Indeed, the use of PSA as a screening biomarker is associated with controversies, particularly since none of the clinical trials that assessed the benefits of PSA included Africans [12]. This underscores the requirement for more effective novel biomarkers for the diagnosis, treatment monitoring, and prediction of PCa outcomes. 

Nucleic acids, such as DNA, can be released into the bloodstream either from living cells via extracellular vesicles or during cell death. Circulating DNA is referred to as cell–free DNA (cfDNA) and has emerged as a promising candidate for diagnosis, prognosis, and treatment monitoring [13,14,15,16,17,18]. In physiological conditions, the amount of cfDNA in blood ranges between 1.7 and 100 ng/mL [19] and between 12 and 439 ng/mL in urine [20]. In cancer patients, the concentration of cfDNA in blood can rise substantially, ranging from 50 to 1000 ng/mL, with tumor DNA constituting 3–90% of the total cfDNA pool [21,22]. The increase in cfDNA levels in cancer patients is attributed to a high turnover rate of tumor cells and ineffective clearing of dead and dying cells [23].

CfDNA carries tumor-specific variations such as mutations in tumor suppressor genes and oncogenes, microsatellite instability, and DNA methylation [24,25,26]. Changes in a single nucleotide in the DNA sequence, such as single nucleotide variants (SNVs), have been detected in the blood of cancer patients. For example, cfDNA in the blood of PCa patients carries somatic alterations, including nonsynonymous variants in cancer-related genes such as *FOXA1*, *ATM*, *PTEN*, and *MED12* [27]. Blood-based cfDNA analysis of the androgen receptor (*AR*) gene can help predict resistance to enzalutamide and abiraterone in metastatic castration-resistant prostate cancer (mCRPC) [28]. Furthermore, the genomic profiling of cfDNA has shed light on differences in the genomic landscape between Caucasian and African American PCa patients, highlighting a higher number of alterations in genes like *AR*, *EGFR*, *MYC*, *FGFR1*, and *CTNNB1* in African American patients [29]. 

In PCa, urine is an important biofluid to consider, given the anatomical location of the prostate gland around the urethra [30]. CfDNA in blood can also cross the kidney barrier to reach the urine [31]. Because urine collection is truly a non-invasive process, it offers an intriguing source for cfDNA [32]. This is especially true in the African context, as urine collection requires only a sterile container, enabling easy collection in remote locations without medical facilities. When comparing DNA methylation in PCa tissue with urinary cfDNA, about 40% of the tumor-specific methylation alterations were found in the urinary cfDNA [30]. When comparing cfDNA mutation patterns between urine and blood, urinary cfDNA had higher mutation frequency, especially in *TP53*, *AR*, *ATM*, *MYC*, and *SPOP* genes [33]. Urinary cfDNA analysis also showed alterations in copy number variations during androgen deprivation therapy (before and after). This included *AR* amplification, *TMPRSS2-ERG* fusion, *PTEN* gene deletion, and *NOTCH1* locus amplification [34]. Together this indicates that urinary cfDNA is a potential source for diagnostic and prognostic marker development.

Given these findings, it becomes imperative to conduct dedicated studies to explore the diagnostic potential of cfDNA in African PCa patients, facilitating more precise management of PCa within the African population.

In this pilot study, we used urinary cfDNA to identify mutation in cancer-related genes in a cohort of South African PCa patients. Genetics study in South Africa are of particular importance due to the distinct ancestral backgrounds present within the population, including Black, Colored, and White populations. Black South Africans primarily trace their lineage to Bantu-speaking populations, enriched by contributions from the KhoeSan people. The Coloured community has a more diverse genetic makeup, originating from the mingling of early European settlers, enslaved individuals from the Dutch East Indies, and indigenous Southern Africans from both Bantu and KhoeSan groups. The White population mainly descends from European settlers. Together, our data reinforce the notion of investigating cfDNA as a non-invasive biomarker for diagnosing PCa in the South African population.

## 2. Materials and Methods

### 2.1. Ethics and Sample Collection

Ethical approval was obtained from the Human Research Ethics Committee of the Faculty of Health Science, University of Cape Town, South Africa (HREC 454/2012). Participation was voluntary, and written consent was obtained from each participant. Urine and tissue samples from patients diagnosed or suspected to have PCa were collected. The participants were recruited from the urological clinics of Groote Schuur and New Somerset Hospitals in Cape Town, South Africa.

Prostate tissue samples were collected from patients during the transurethral resection of the prostate (TURP) surgical procedures. Around five tissue samples (chips) were collected in RNAlater^®^ (Waltham, MA, USA) and transported to the laboratory. The tissue chips were cut longitudinally in half, one part was stored in RNAlater, and the second was formalin-fixed and paraffin-embedded (FFPE). The FFPE tissue blocks were sectioned at 5 μm and stained with hematoxylin and eosin (H&E) to identify the tumor and normal area in each block. Histopathologic evaluation was performed to assess the percentage of tumor and the Gleason score of each tissue sample by the South African National Health Laboratories. The urine was collected before any interventions and centrifuged at 1000× *g* for 10 min to remove cell debris.

### 2.2. CfDNA Isolation and Quantification from Urine

cfDNA was isolated from 10 mL of urine using QIAamp Circulating Nucleic Acid Kit (Hilden, Germany, Catalog number: 55114) using a vacuum manifold. In short, 10% Proteinase K was added to ATL buffer, ACL buffer, and urine in a 1:4:4 ratio to digest the urinary proteins. The solution was incubated for 30 min at 60 °C before 1 volume (the total volume) of ACB buffer was added. After mixing, it was incubated for 5 min on ice. The solution was applied to the QIAamp mini-column setup in a QIAvac 24 Plus and filtered with a vacuum. The column was washed in three steps with 600 µL of ACW1 buffer, 750 µL of ACW2 buffer, and 750 µL of 100% ethanol. After a 10 min, 56 °C drying step, the cfDNA was eluted in 50 µL of nuclease-free water and stored at −80 °C.

### 2.3. Whole Exome Sequencing from Urinary cfDNA

Whole-exome sequencing was performed using cfDNA extracted from 12 urine samples. The concentration and fragment size of the cfDNA were determined using the Agilent 2100 Bioanalyzer (Agilent Technologies, Inc., Santa Clara, CA, USA; Catalog Number G2939BA) with the Agilent High Sensitivity DNA chip (Agilent Technologies, Inc., Santa Clara, CA, USA; Catalog Number 5067-4626). The whole exome sequencing required 20 ng of cfDNA with a fragment size between 150 and 200 bp. Whole exome sequencing libraries were constructed with 20 ng of cfDNA using the SureSelect Human All Exon V6 kit (Agilent Technologies, Inc., Santa Clara, CA, USA; Catalog Number 5190-8863). Paired-end (150 bp) sequencing was performed on the Novaseq 6000 (Agilent Technologies, Inc., Santa Clara, CA, USA). The sequencing statistics are provided in Supplementary Table S1.

### 2.4. DNA Isolation from Tissue and Buffy Coat

Tumor tissue with a purity higher than 80% was used for DNA isolation as the tumor sample. Normal tissue or, alternatively, buffy coat was used for DNA isolation as the normal sample. The genetic material was extracted from 30 mg of tissue with the Qiagen AllPrep DNA/RNA isolation kit following the manufacturer’s recommendations. 

### 2.5. Whole-genome sequencing from PCa Tissue

The quality and quantity of the isolated DNA was determined using a NanoDrop™ One/OneC (Thermo Fisher, Waltham, MA, USA; Catalog ND-ONEC) and Agilent 2100 Bioanalyzer (Agilent Technologies, Inc., Santa Clara, CA, USA). For whole-genome sequencing (WGS), the genomic DNA was fragmented using Covaris technology and a 350 bp fragment was selected. Whole genome paired-end sequencing was performed using the BGISEQ-500 platform (BGI, Shenzhen, China). The sequencing statistics are provided in Supplementary Table S2.

### 2.6. Data Analysis

Data analysis for our cfDNA whole exome sequencing data was performed using an in-house developed pipeline. The pipeline involves a preprocessing stage of de-multiplexing the raw data originally in a BCL file using the bcl2fastq tool into separate FASTQ files for each of the samples. The FASTQ files were then filtered using the AfterQC [35] tool optimized for cfDNA data to remove reads of low quality. The filtered FASTQ files were then aligned to the reference genome with the help of Burrows–Wheeler Aligner (BWA) version 0.7.17 [36]. The SAM file generated after alignment was changed into BAM and then indexed using Samtools. Next, we removed duplicate reads using the Samtools rmdup tool. After the BAM file processing was completed, we performed variant calling for somatic mutation using VarScan2 [37]. The VarScan2 caller has previously been used with good success in previous cfDNA analysis. The VCF file generated after the calling was annotated with Catalogue of Somatic Mutations In Cancer (COSMIC) database version 95 using ANNOVAR with build hg19 databases. Then, the false-positive mutations were marked using MySQL baseline technology, after which the VCF filtered and cleaned. The cleaned VCF file was used in reporting the final target mutations. 

Data analysis was performed using the BGI pipeline. Raw reads were filtered to remove sequencing adapters, read with low quality ratio in more than 50% of the bases (base quality less than or equal to 5) and reads with more than 10% unknown bases. After filtering, the reads were mapped to the human reference genome (GRCh37/HG19) using BWA [38]. To ensure the precision of variant calling, we adhered to the recommended best practices for variant analysis with the Genome Analysis Toolkit (GATK), available at https://www.broadinstitute.org/gatk/guide/best-practices (accessed on 15 April 2023). GATK was employed for local realignment around insert and deletion region and base quality score recalibration, while duplicate reads were eliminated using Picard tools.

Genomic variations, encompassing SNVs, were identified utilizing HaplotypeCaller from GATK (v3.3.0). Subsequently, the variant quality score recalibration (VQSR) method, leveraging machine learning to pinpoint annotation profiles of credible variants, was employed to yield high-confidence variant calls.

### 2.7. Statistical Analysis

A *t*-test was used to compare the number of SNVs in each gene between the benign prostatic hyperplasia (BPH) and PCa patients. A *p*-value lower than 0.05 was considered statistically significant. The heatmap was generated using the function pheatmap from the homonym R package. Ward’s linkage was chosen as method for the hierarchical clustering.

## 3. Results

### 3.1. Cell-Free DNA Whole Exome Sequencing

We isolated the cfDNA from urine samples donated by four patients with BPH and eight patients with PCa. The participants were selected to have a cohort that fully represented the heterogeneity of clinical presentation of South African patients. Patients with aggressive disease and PSA over 100 ng/mL were included. The patients’ clinical data are shown below in Table 1.

We performed the whole-exome sequencing of the cfDNA isolated from the urine samples. We identified SNVs that could contribute to population-specific routes of tumorigenesis in South African patients (Supplementary Table S3). We identified a total of 2,947,228 SNVs across all samples, of which 10.6% was in a coding region. These are all differences detected from the alignment with the human reference genome (i.e., hg19), including somatic and germline population-specific mutations. In order to identify the mutations associated with cancer, we filtered all exonic SNVs to select only those with a reported association with cancer using COSMIC. A total of 99,923 nsSNVs associated with cancer across all samples were identified. Interestingly, 7198 are associated specifically with PCa and are located in 1078 different genes.

**Figure 1:**
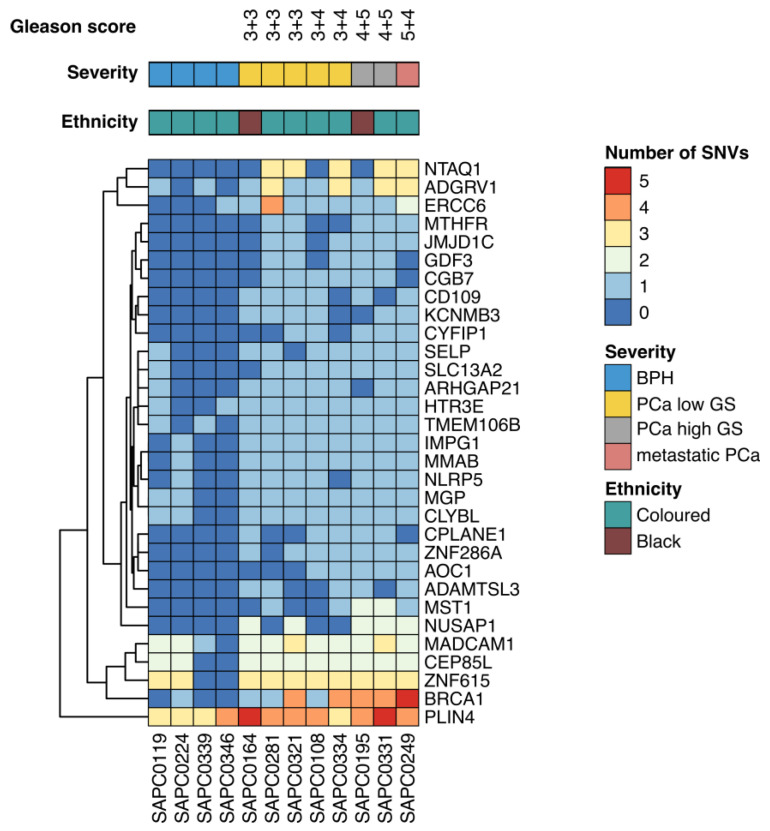
Heatmap of significant mutated gene between BPH and PCa patients colored according to the number of mutations found in each of the samples. [GS-Gleason score].

The mutated genes identified were analyzed according to the frequency of their occurrence in PCa and BPH samples. Remarkably, we found 31 genes showing a statistically significant difference in the presence of SNVs (Figure 1).

### 3.2. Identification of Frequently Mutated Genes Associated with PCa

Next, we sought to focus our analysis on those genes among the 31 identified during the sequencing that have been previously associated with PCa in African men. Two genes involved in DNA damage repair/response (i.e., *BRCA1* and *ERCC6*) have been reported to be frequently mutated in African Americans compared to European Americans [39,40]. 

*ARHGAP21*, a negative regulator of cancer cell growth, migration, and invasion, and *ADAMTSL3*, essential for the development and growth of lymphatic vessels, have been reported to have increased methylation levels in high-Gleason-score PCa in African Americans [41]. We investigated the incidence of the SNVs found in these four genes across all cfDNA samples. In order extend our investigation to an additional number of patients, we retrieved the information of the SNVs from whole-genome sequencing analysis of 11 patients. The clinical and demographics features are shown in Table 2.

**Figure 2:**
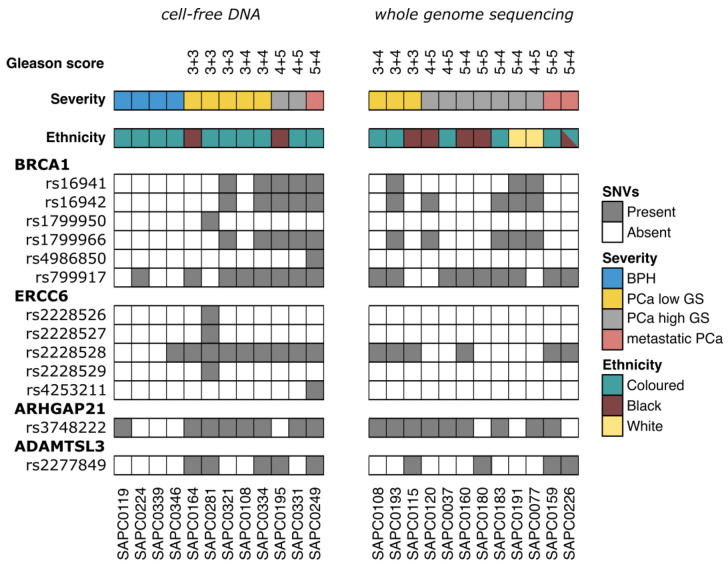
Occurrence of SNVs associated with PCa in *BRCA1*, *ERCC6*, *ARHGAP21*, and *ADAMTSL3*.

All SNVs were identified both in tumor and normal tissue, suggesting their germline origin (Figure 2). We observed that the SNV in *BRCA1* with the highest incidence in our cohort was rs799917. In the cfDNA cohort, rs799917 was present in 7/8 (87.5%) patients with PCa, while its incidence in BPH patients was only one in four patients (25%). In the whole-genome sequencing cohort, rs799917 was present in 9/12 (75.0%) patients with PCa. Interestingly, rs799917 has higher incidence (81.3%) in Coloured (13/16) compared to Black (4/6) ethnicity, slightly higher than the value of 68% reported in the African American population [42]. A higher incidence of rs16941, rs16942, and rs1799966 was observed in Coloured and White ethnicities compared to the Black ethnicity.

The analysis in the other genes demonstrated that, in *ERCC6*, the SNV with the highest incidence in our cohort was rs2228528. Interestingly, it is associated with 100% patients with PCa, 25% of the patients with BPH in the cfDNA cohort, and 50% of the patients with PCa in the whole-genome sequencing cohort. Finally, analysis of the other two PCa-related genes found them to be linked to one SNV each: *ARHGAP21* (rs3748222) and *ADAMTSL3* (rs2277849). rs3748222 is associated with 7/8 (87.5%) of patients with PCa and 1/4 (25%) of patients with BPH in the cfDNA cohort and 75.0% of the patients with PCa in the whole-genome sequencing cohort. rs2277849 is associated with 5/8 (62.5%) of patients with PCa and none of the patients with BPH in the cfDNA cohort and 33.3% of the patients with PCa in the whole-genome sequencing cohort.

## 4. Discussion

CfDNA released from tumor cells is a mirror of the ongoing state of the tumor and measuring cfDNA level can serve as an ideal diagnostic and prognostic tool for tumors [43]. To determine genetic variation within the cfDNA fragments in PCa from South African population, we performed exome sequencing of urinary cfDNA. Our study is the first African-based whole exome sequencing of urinary cfDNA in PCa and helps to provide better understanding of the specific genetic alteration in PCa among African populations. By sequencing the urinary cfDNA of eight PCa samples and four BPH samples, we identified 31 significantly mutated genes. 

Our finding of this novel panel of mutated genes in the PCa of South African men greatly contributes towards the effort of identifying genetic mutations specific to the African population. These mutated genes may potentially be employed as PCa diagnostic biomarkers particularly in the African population. Also, these findings contribute towards the search of more specific liquid biopsies for African men considering that the presently available liquid biopsies for PCa diagnosis were based on studies performed among Caucasian populations [44,45]. 

Among the significantly mutated genes, four (*BRCA1*, *ERCC6*, *ARHGAP21*, and *ADAMTSL3*) were previously described to be mutated in PCa of the African population. The role of the *BRCA1* gene in the development of PCa has been extensively described [46,47,48,49]. *BRCA1* is a tumor suppressor gene inherited in an autosomal dominant fashion with incomplete penetrance. The development of tumors in patients with germline mutations in *BRCA1* genes requires somatic inactivation of the remaining wild-type allele [48]. BRCA1 plays a major role in cellular control systems due to its role in different cellular processes such as transcriptional regulation, DNA damage response and repair, and chromatin modeling [50,51]. This gene has been shown to be a coregulator of AR, mediating important signaling pathways in prostrate carcinogenesis and progression [52,53]. Mutations in *BRCA1* and *BRCA2* genes are linked with poor prognosis of PCa [47,54,55]. Studies have shown a higher *BRCA1* mutation in PCa in men of African ancestry than the Caucasian populations [39,40,56,57]. 

The excision repair cross-complementing group 6 (*ERCC6*) gene encodes a protein that plays a key role in the repair of damaged DNA [58,59]. ERCC6 functions through transcription and nucleotide excision repair (NER), which works by removing bulky adducts and repairing DNA damage produced by environmental agents such as ultraviolet light [60]. *ERCC6* gene mutation can reduce its activity, thereby leading to defects in the NER repair of damaged DNA. Additionally, the *ERCC6* gene is more frequently mutated in the PCa of African American populations [39]. 

ARHGAP21 belongs to the RhoGAP protein family, which plays a major role in the conversion of Rho-GTPases from an active to inactive bound state [61,62]. Rho family GTPases are involved in the regulation of several cell functions, such as cell adhesion, migration, proliferation, and survival [63]. ARHGAP21 plays major functions in cell–cell interaction, vesicular trafficking of Golgi membranes, and cardiac stress [61,64,65]. Studies have reported that ARHGAP21 is a negative regulator of cancer cell growth, migration, and invasion [66,67,68]. Low expression of ARHGAP21 has been shown to correspond with worse prognosis in prostate, lung, ovarian, and colon cancer [68,69,70,71]. Interestingly, it was the diagnostic potential of the *ARHGAP21* gene in African American PCa patients was also reported [41]. ADAMTSL3 is one of the superfamilies of cell-surface-associated glycoproteins comprising nineteen ADAMTS proteases and seven ADAMTS-like (ADAMTSL) proteins [72]. ADAMTS proteases play a major function in biological processes including procollagen maturation, connective tissue assembly, angiogenesis, and cancer [73,74,75]. ADAMTSL proteins do not directly participate in proteolytic activity; they are majorly involved in the regulation of ADAMTS activity and assembly of extracellular matrices [72,76,77]. The proliferative role of ADAMTSL3 has been described in different human cancers [78,79,80]. In addition, ADAMTSL3 was also reported as a putative diagnosis marker of PCa in Africans [41].

Previous studies have shown the potential role of cfDNA concentration and DNA integrity as diagnostic and prognostic biomarkers for PCa, which is consistent with our findings. Our findings on the genetic profiling of cfDNA in South African patients help contribute to the possibility of finding genetic mutations specific to the African population known for aggressive PCa disease. This will greatly help in developing a population-specific biomarker in the diagnosis of PCa in the South African population. Our study also supports an earlier postulation made that the combination of PSA and cfDNA serves as a more specific and sensitive biomarker in the diagnosis of PCa. While we emphasize the necessity for further studies, particularly due to the limited number of patients in our cohort, this is, to our knowledge, the first report on cancer-associated SNVs in urinary cell-free DNA among South African men with PCa.

## 5. Conclusions

Our study focused on utilizing cfDNA whole exome sequencing to investigate the genetic landscape of PCa in a diverse South African patient population. Interestingly, we identified SNVs located within coding regions as well as cancer-related SNVs. The analysis of mutated genes revealed a panel of genes statistically different between BPH and PCa patients. Notably, four genes (*BRCA1*, *ERCC6*, *ARHGAP21*, and *ADAMTSL3*) were previously associated with PCa in Africans. These genes play crucial roles in various cellular processes, and their mutations have been linked to the development and progression of PCa. The prevalence of specific SNVs varied across different patient groups, including those with high Gleason scores and metastatic disease. These findings underscore the potential clinical relevance of these genetic markers, especially for aggressive forms of PCa. Furthermore, our study sheds light on the importance of considering genetic diversity in developing diagnostic and prognostic biomarkers, as existing liquid biopsy approaches have been predominantly based on studies on Caucasian populations. In summary, our research advances our understanding of the genetic underpinnings of PCa in the South African context. The identified mutated genes and associated SNVs hold promise as potential diagnostic and prognostic biomarkers for PCa, particularly in African populations.

## Figures and Tables

**Table 1:** Clinical data of patient samples used for whole exome sequencing.

Patient ID	Age (Years)	Ethnicity	Pathology	Gleason Score	PSA (ng/mL)
SAPC0119	69	Coloured	BPH		5.5
SAPC0224	81	Coloured	BPH		14.1
SAPC0339	77	Coloured	BPH		12.9
SAPC0346	69	Coloured	BPH		1.1
SAPC0164	74	Black	PCa	3 + 3	74
SAPC0281	71	Coloured	PCa	3 + 3	19
SAPC0321	63	Coloured	PCa	3 + 3	8.6
SAPC0108	56	Coloured	PCa	3 + 4	82.6
SAPC0334	73	Coloured	PCa	3 + 4	126.8
SAPC0195	87	Black	PCa	4 + 5	26
SAPC0331	75	Coloured	PCa	4 + 5	53.2
SAPC0249	63	Coloured	Metastatic PCa	5 + 4	1070

**Table 2:** Clinical data of patient samples used for whole genome sequencing.

Patient ID	Age (Years)	Ethnicity	Pathology	Gleason Score	PSA (ng/mL)
SAPC0108	56.6	Coloured	PCa	3 + 4	96.3
SAPC0193	63.5	Coloured	PCa	3 + 4	48.85
SAPC0115	82.0	Black	PCa	3 + 3	41
SAPC0120	82.3	Black	PCa	4 + 5	289.9
SAPC0037	66.9	Coloured	PCa	4 + 5	27
SAPC0160	59.2	Black	PCa	5 + 4	738
SAPC0180	58.1	Black	PCa	5 + 5	5000
SAPC0183	63.9	Coloured	PCa	5 + 4	34.2
SAPC0191	61.8	White	PCa	5 + 4	576
SAPC0077	71.7	White	PCa	4 + 5	3.83
SAPC0159	75.2	Coloured	Metastatic PCa	5 + 5	5000
SAPC0226	70.3	Coloured/Black	Metastatic PCa	5 + 4	332.3

## Data Availability

All data generated and analyzed during the current study are available in the Supplementary Table file. Any remaining data can be obtained from the corresponding author upon reasonable request.

## Supplementary Materials

The following supporting information can be downloaded at: https://www.mdpi.com/article/10.3390/genes14101884/s1, Supplementary Table file, containing: Supplementary Table S1: Sequencing statistics of cfDNA analysis; Supplementary Table S2: Sequencing statistics of whole genome analysis; Supplementary Table S3: Summary of the number of SNVs found in cfDNA.
